# Topical exposure to triclosan inhibits Th1 immune responses and reduces T cells responding to influenza infection in mice

**DOI:** 10.1371/journal.pone.0244436

**Published:** 2020-12-29

**Authors:** Hillary L. Shane, Sreekumar Othumpangat, Nikki B. Marshall, Francoise Blachere, Ewa Lukomska, Lisa M. Weatherly, Rachel Baur, John D. Noti, Stacey E. Anderson

**Affiliations:** Allergy and Clinical Immunology Branch, Health Effects Laboratory Division, National Institute for Occupational Safety and Health, Centers for Disease Control and Prevention, Morgantown, WV, United States of America; University of Michigan Health System, UNITED STATES

## Abstract

Healthcare workers concurrently may be at a higher risk of developing respiratory infections and allergic disease, such as asthma, than the general public. Increased incidence of allergic diseases is thought to be caused, in part, due to occupational exposure to chemicals that induce or augment Th2 immune responses. However, whether exposure to these chemical antimicrobials can influence immune responses to respiratory pathogens is unknown. Here, we use a BALB/c murine model to test if the Th2-promoting antimicrobial chemical triclosan influences immune responses to influenza A virus. Mice were dermally exposed to 2% triclosan for 7 days prior to infection with a sub-lethal dose of mouse adapted PR8 A(H1N1) virus (50 pfu); triclosan exposure continued until 10 days post infection (dpi). Infected mice exposed to triclosan did not show an increase in morbidity or mortality, and viral titers were unchanged. Assessment of T cell responses at 10 dpi showed a decrease in the number of total and activated (CD44^hi^) CD4+ and CD8+ T cells at the site of infection (BAL and lung) in triclosan exposed mice compared to controls. Influenza-specific CD4+ and CD8+ T cells were assessed using MHCI and MHCII tetramers, with reduced populations, although not reaching statistical significance at these sites following triclosan exposure. Reductions in the Th1 transcription factor T-bet were seen in both activated and tetramer+ CD4+ and CD8+ T cells in the lungs of triclosan exposed infected mice, indicating reduced Th1 polarization and providing a potential mechanism for numerical reduction in T cells. Overall, these results indicate that the immune environment induced by triclosan exposure has the potential to influence the developing immune response to a respiratory viral infection and may have implications for healthcare workers who may be at an increased risk for developing infectious diseases.

## Introduction

Healthcare workers encounter a number of health hazards in the workplace, making the healthcare profession one of the top hazardous environments for workers [1, 2]. One of these hazards is the exposure to chemicals, often in the form of antimicrobials of a variety of classes that have been linked to the development of allergic diseases [3]. Triclosan (2,4,4′-trichloro-2′-hydroxydiphenyl ether; TCS) is an antimicrobial compound that has been used in a variety of consumer products including soaps and hand sanitizers for decades [4–6] until a recent ruling by the FDA that banned TCS in many consumer products, although it is still used in clinical formulations [7]. TCS is absorbed through the skin, and increased levels of urinary TCS was found in healthcare workers from hospitals that use triclosan containing hand soaps [8]. Most commercially available products contained TCS at levels from 0.1%-0.45%, while antimicrobials used in clinical settings can have TCS levels up to 1% wt/vol [9]. TCS has been associated with many negative health effects, but one of the more well studied aspects is TCS’s promotion of allergy-like symptoms associated with TCS exposure. Associations between TCS exposure and a variety of allergic diseases have been identified, including hay fever, and the exacerbation of food and aeroallergens [10–13]. Previous work in our laboratory has found that although not directly sensitizing [14], topical application of TCS augments the allergic response to OVA in a murine model of asthma [15]. Further investigation revealed that TCS’s augmentation of the allergic response occurs through a thymic stromal lymphopoietin (TSLP) mediated pathway, which results in the exacerbation of Th2 immune responses including the production of IL-4, IL-13 and the Th2 transcription factor GATA3 [16]. Despite these health effects, and evidence of relevant occupational exposure [8], TCS is still used in select antimicrobial agents used in clinical settings [17].

Another hazard that healthcare workers regularly encounter is occupational exposure to infectious diseases; it is estimated that between 13–42 healthcare workers per million die annually from occupationally acquired infections [1]. Due to their close contact with potentially infected patients, healthcare workers may have an increased risk of contracting infectious diseases spread by airborne transmission, including influenza [18]. A review of the literature identified influenza as the most commonly reported single pathogen responsible for outbreaks in long term care facilities, causing ~24% of reported outbreaks, and that health care workers are at increased risk of infection during an outbreak [19]. This is particularly true during pandemics, for example, during the 2009 A(H1N1)pdm09 influenza pandemic healthcare workers had an increased risk of infection (OR = 2.08) compared to control populations [20], with physicians and nurses having a particularly high risk (OR = 5.25) [21]. While vaccination is thought to be an effective method of protection against influenza infections in healthcare settings, and healthcare worker compliance in the United States is high with a 78.4% overall coverage rate during the 2017–2018 flu season [22], studies focusing specially on influenza vaccine efficacy in health care workers are limited and difficult to draw conclusions from [23]. Furthermore, these seasonal vaccinations do not provide protection against emerging pandemic strains. Additionally, certain occupations within “healthcare worker” category such as assistants and aides and non-clinical support staff (including housekeeping, maintenance staff, janitors, and laundry workers) have lower rates of coverage [22]. Due to the increased susceptibility of healthcare workers to contracting infectious diseases, especially those transmitted by the respiratory route (such as influenza), it is important to identify the potential effects that occupational chemicals have on the development of protective immune responses to these pathogens.

Immunological defense against intracellular pathogens, including influenza viruses, includes the development of Th1-polarized cell populations. This polarization is guided by the master transcription factor T-bet and is characterized by the production of Th1 cytokines (largely IFN-γ) [24]; T-bet also plays an important role in the generation of functional effector CD8+ T cells [25]. Both CD4+ and CD8+ T cells play an important role in the clearance of respiratory viral pathogens, including influenza [26, 27]. During infection, effector T cells migrate into the lung and lung airways where they produce cytokines that promote Th1 responses and eliminate influenza infected respiratory epithelial cells through cytotoxic mechanisms [28]. In regards to CD4+ T cell responses, Th1 polarized influenza specific CD4+ T cells can transfer immunity to a naïve host, while Th2 polarized cells offer no protection [29], highlighting the need for the induction of proper immunological responses. Previous studies, in multiple infectious disease models, have shown that parasitic infections that skew the immune environment towards Th2 responses can negatively impact the development of appropriate Th1 immune responses against secondary pathogens [30]. As TCS induces a Th2 polarized environment [16] and can augment allergic responses that develop in the lung [15] following topical exposure, it raises the question if appropriate antiviral T cell responses, which depend on Th1 polarization, will develop properly in a TCS exposed individual. In this manuscript, we tested the hypothesis that the Th2-promoting immune environment induced by topical exposure to the antimicrobial chemical TCS will impair the Th1-mediated T cell responses to influenza infection in a murine model.

## Materials and methods

### Chemical exposures

Mice were dosed daily with acetone (VC: vehicle control) (Sigma-Aldrich; CAS # 67-64-1) or 2% triclosan (TCS) (Calbiochem; CAS #: 3380-34-5) diluted in acetone, starting at study day -7 through 10 days post infection (dpi). Chemicals were applied on the dorsal surface of each ear in a 25 μl/ear volume. Timing and concentration of TCS dosing was determined based off of previous studies for augmenting Th2 immune responses [16].

### Animals

Female BALB/c mice (6–8 weeks-of age) were purchased from Taconic. This strain has previously been used in our laboratory to thoroughly characterize immune responses following topical exposure to TCS [14–16, 31]. Each shipment of animals was randomly assigned to a treatment group, weighed and individually identified (via tail marking) using a permanent marker and acclimated for a minimum of five days upon arrival. The animals were housed at a maximum of five per cage in ventilated plastic shoebox cages with hardwood chip bedding. NIH-31 modified 6% irradiated rodent diet (Harlan Teklad) and tap water were provided from water bottles, *ad libitum*. The animal facility temperature was maintained at 68–72° F and the relative humidity between 36–57%. A light/dark cycle was maintained on 12-hour intervals. All animal experiments were performed in the AAALAC International accredited NIOSH animal facility in accordance with an animal protocol approved by the CDC Morgantown Institutional Animal Care and Use Committee.

### Intranasal influenza infections

**Fig 1:**
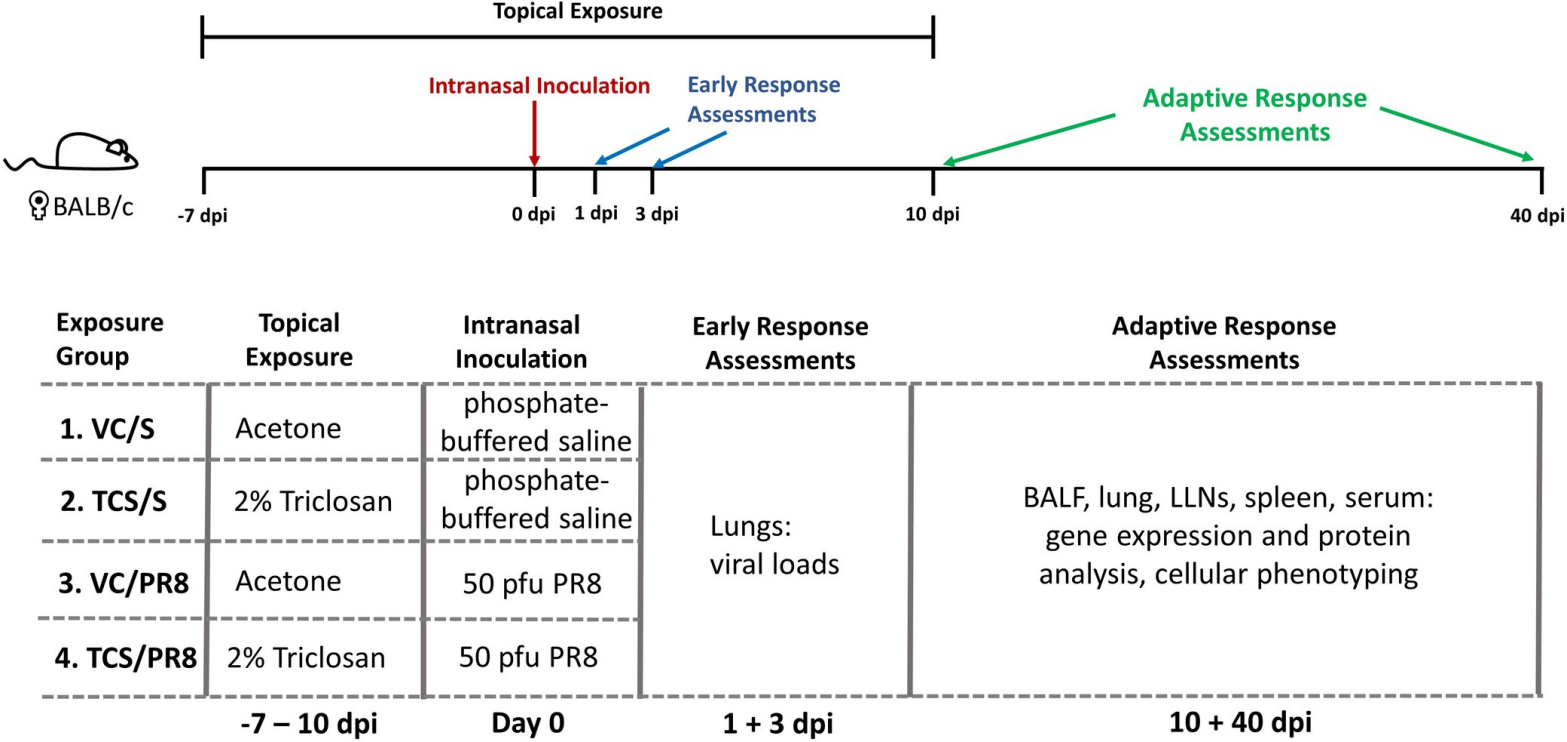
Diagram of experimental scheme. BALB/c mice were dermally dosed with acetone or 2% TCS on the dorsal surface of the ear (25 μl/ear) starting at day -7 and continuing through 10 days post infection (dpi). On Day 0, mice were intranasally inoculated with 50 pfu of Influenza A virus PR8 (PR8) diluted in saline or mock infected with saline in a 50 μl volume. Mice were sacrificed on days 1 and 3 post infection to assess viral titers and at days 10 and 40 post infection to assess adaptive immune responses as noted. Exposure groups contained 5 mice/group per timepoint.

Mouse-adapted A(H1N1) influenza A/Puerto Rico/8/34 (PR8) was generously provided by Dr. Gary Burleson (Burleson Research Technologies, Inc.) and propagated in the allantoic cavity of SPF Research Grade Fertile Chicken Egg Flock R128 (Charles River) [32]. A dose finding study (30 mice over a 14-day infection period) was performed to determine a sub-lethal dose (50 plaque forming units (pfu)) that was used in the studies presented in this manuscript (S1 Fig). Following infection, mice were monitored for weight-loss and other signs of morbidity (e.g., hunched posture, respiratory stress, or other abnormal behaviors) throughout the course of infection at least every 24 hours; mice were monitored every 12 hours between once weight loss was recorded and symptoms started to be observed. Mice were euthanized if weight loss exceeded 20% and mice showed signs of respiratory stress. In the range finding experiment 8 mice met this criteria for euthanasia, and were humanely euthanized prior to the end of the study by injection of a sodium pentobarbital-based solution. For infections, virus was diluted in USP phosphate buffered saline (S) in order to obtain a dosage of 50 pfu/50 μl. Virus or S was instilled intranasally in mice that were anesthetized with isoflurane. Mice were monitored following infection for recovery from anesthesia and infection as described above. The combined chemical exposures and influenza infections resulted in a total of 40 mice in four treatment groups per timepoint (10 days of infection and 40 days of infection) (Acetone + S [VC/S], triclosan + S [TCS/S], Acetone + PR8 [VC/PR8] and triclosan + PR8 [TCS/PR8]) as outlined in Fig 1.

### Tissue collection and processing

Animals were humanely euthanized by injection of a sodium pentobarbital-based solution on the indicated dpi. Lungs collected for plaque assays were excised and placed in a sterile 2 ml snap cap tube and immediately frozen in liquid nitrogen. Snap-frozen tissues were stored at -80°C until used. Cells and proteins from the lung airways were obtained by means of bronchoalveolar lavage (BAL) in which the trachea was intubated, and 1 ml S was introduced and recovered from the airways. The first fraction of BAL was kept separate, while the 2^nd^ and 3^rd^ washes were combined. The first BAL fraction was centrifuged to pellet cells (5 minutes at ~750 x g) and the supernatant (BAL fluid; BALF) was collected and stored at -20°C to be used for protein analysis. The cellular portion of the first fraction was combined with the others collected to use for cellular phenotyping. Lungs, perfused with S, were collected into RPMI and kept on ice. Lungs were manually chopped with scissors into small pieces and digested at 37°C for one hour in a digestion media containing 0.125 mg/ml Liberase TL (Roche) and 50 ug/ml DNase 1 (Sigma) in RPMI. Digestion was stopped by the addition of RPMI/10% FBS. The lung tissue was then mechanically disrupted on a gentleMACS dissociator (Miltenyi Biotech) then filtered through a 70 μm nitex filter to obtain a single cell suspension. Lung associated lymph nodes (LLNs) and spleens were collected into RPMI, then mechanically disrupted between the frosted ends of two microscope slides and filtered through a 70 μm pore filter to obtain a single cell suspension. Cells were centrifuged (5 minutes at ~750 x *g*) and resuspended before counting. LLNs and spleen cells were counted using a coulter counter (Beckman Coulter) following RBC lysis with Zapoglobin lytic reagent (Beckman Coulter). BAL and lung cells were counted on a Cellometer (Nexcelom) using AO/PI solution (Nexcelom) to determine numbers of live cells.

### Lung homogenization

For viral plaque assay (VPA) analysis, excised lung tissue was homogenized using a TissueLyser II (Qiagen). Briefly, 50 mg of thawed lung tissue was placed in a 2.0 ml microtube containing 500 μl cold Hank’s Balance Salt Solution (HBSS; ThermoFisher Scientific) supplemented with 0.1% bovine serum albumin (BSA; Sigma-Aldrich), 100 units/ml penicillin G and 100 units/ml streptomycin (ThermoFisher Scientific), and one 5 mm stainless steel bead (Qiagen). Sample tubes were loaded into a cold TissueLyser II rack and homogenized at 30 Hz for 3 minutes. Homogenized lung tissue samples were serially diluted in supplemented HBSS and stored on ice until VPA.

### Viral plaque assay

Madin-Darby Canine Kidney (MDCK) cells (ATCC) cultured to ~90% confluence were detached with 0.25% Trypsin-EDTA (ThermoFisher Scientific), washed and re-suspended in complete Eagle’s Minimum Essential Medium (EMEM; ATCC) at a density of 1.0 x 10^6^ cells/ml. Next, 2.0 ml of the cell suspension was added to each well of a 6-well CoStar tissue culture plate (Corning) and incubated overnight at 35°C in a humidified 5% CO_2_ incubator. Confluent cell monolayers were washed twice with 2.0 ml of 1X S, inoculated with 400 μl of diluted, homogenized lung tissue and incubated for 45 min at 35°C in a humidified 5% CO_2_ incubator. Inoculated cells were then washed once with 2.0 ml of 1X S, overlaid with supplemented Dulbecco’s modified Eagle’s medium (DMEM)/F12 containing 100 units/ml penicillin G/100 μg/ml streptomycin (ThermoFisher Scientific), 2 mM l-glutamine (ThermoFisher Scientific), 0.2% BSA (Sigma–Aldrich), 10 mM HEPES (ThermoFisher Scientific), 0.22% sodium bicarbonate (ThermoFisher Scientific), 0.01% DEAE-dextran (MP BioMedicals, LLC, Solon, OH), 0.6% agarose (Oxoid Ltd.) and 2 μg/ml N-p-tosyl-l-phenylalanine chloromethylketone (TPCK; Sigma–Aldrich), and incubated at 35°C for 52-hours in a humidified 5% CO_2_ incubator. The cells were then fixed with 2.0 ml of 10% formalin for 15 minutes and the agarose overlay was removed by washing with tap water. Plaques were stained with 2.0 ml of 1% crystal violet/0.19% methanol for 15 minutes, rinsed with tap water, dried and plaque forming units (PFUs) were calculated.

### TCID50/HA assay

Influenza virus titers in mouse lung tissues were determined using the tissue culture infective dose 50% (TCID50) combined with hemagglutination (HA) assay. Mouse lungs were placed in 2 ml of ice-cold S and homogenized using gentleMACS Dissociator (Miltenyi Biotec GmbH) and centrifuged at 600 g for 10 minutes. Supernatant was collected and used for the TCID/HA assay. Ten-fold serial dilutions of the supernatants were prepared using Minimal Essential Medium (MEM, ATCC) containing 2 μg/ml TPCK. MDCK (1.0×10^5^ cells/mL) cells were plated in a U-bottom 96-well plate (Corning). The diluted supernatants were added to the 96 well plate and then incubated overnight at 37°C/ 5% CO_2_ incubator. The next day, the medium was replaced with fresh MEM. After 72 hours of incubation, 50 μl of 0.5% turkey RBCs in S was added to each well. The plates were then incubated for 1 h at 4°C and the HA patterns were read to determine the TCID50. A positive (PR8) and corresponding negative controls were added in each plate. Hazy wells show turkey erythrocytes agglutination while non-agglutinated wells show a distinct RBC “button” at the bottom of the well. TCID50 was then calculated by the Reed-Muench formula [33, 34].

### Gene expression analysis

An aliquot of cells from the single cell preparation of lung tissue was resuspended in Qiazol (Qiagen) and stored at -80°C until analysis. RNA was extracted using a RNeasy kit according to the manufacturer’s directions (Qiagen) on an automated RNA isolation machine (QIAcube, Qiagen). The RNA concentration and purity were determined using a NanoDrop Spectrophotometer (Thermo Scientific). The cDNA was prepared on an Eppendorf Mastercycler using Applied Biosystems’ High Capacity Reverse Transcription kit. The cDNA was used as template for real-time PCR (RT-PCR) reactions containing TaqMan PCR Master Mix with gene-specific primers (Applied Biosystems) on a 7500 RT-PCR System. Relative fold changes in gene expression were determined using the 2^-ΔΔCT^ method compared to the VC/S group. Data were normalized for expression of housekeeping gene *Actb*. Genes assessed included: *Ifng*, *Tbet*, *Il4*, *Cxcr3*, *Foxp3*, *Lag3*, *Gata3*, *Tnf*, *Ifnar1*, and *Tslp*.

### Luminex assay

Cytokines were measured in 50 μl of BAL fluid using the Th1/Th2/Th9/Th17/Th22/Treg Cytokine 17-Plex Mouse ProcartaPlex™ Panel (ThermoFisher) according to manufacturer’s instructions and data was acquired using a Luminex 200 system (Millipore).

### Flow cytometry

The influenza A HA_143-155_ MHC II (I-A(d)/ HNTNGVTAACSHE) tetramer (APC conjugated) was generated at the National Institute of Allergy and Infectious Diseases Tetramer Facility (Emory University, Atlanta, GA). The influenza A NP_147-155_ MHC I (H-2K(d) /TYQRTRALV) tetramer (PE conjugated) was purchased from MBL International (Woburn, MA). Due to constraints on cell numbers flow cytometry was only performed on LLNs collected from infected mice. Single cell suspensions were spun down and resuspended in 50 μl of RPMI + 5% FBS containing FC Block (anti-mouse CD16/32 antibody [BD Biosciences]) and 20 μg/ml of the influenza A HA_143-155_ MHC II (I-A(d)/ HNTNGVTAACSHE) tetramer (CD4-Tet) and incubated at 37°C for 1 hour. Following the incubation, a cocktail containing the Influenza A NP_147-155_ MHC I (H-2K(d)/TYQRTRALV) tetramer (CD8-Tet) and remaining cellular surface stains in FACS buffer was added (50 μl/sample) and cells were stained for an additional 30 minutes at RT. The fluorochrome-conjugated anti-mouse antibodies included in the remaining surface-staining cocktail were: CD8-Tet-PE, CD4-BV605 (GK1.5, Biolegend), CD8-V500 (53–6.7, BD Bioscience), CD45-eF450 (30-F11), and CD44-APC-eF780 (IM7). Following surface staining, the cells were washed and intracellular staining was carried out using the FoxP3/Transcription Factor Staining Buffer Set (eBioscience/ThermoFisher) according to manufacturer’s instructions. The fluorochrome conjugated antibodies used to stain transcription factors were purchased from Invitrogen: GATA3-FITC (TWAJ) and T-bet-PE-Cy7 (eBio4B10). Appropriate staining controls were included for all tissues including FMO controls, and a human CLIP peptide control (I-A(d) PVSKMRMATPLLMQA) tetramer (APC conjugated) for the CD4-Tet staining (National Institute of Allergy and Infectious Diseases Tetramer Facility, Emory University, Atlanta, GA). Following intracellular staining cells were analyzed immediately on a BD LSRII Flow Cytometer. Flow cytometry data was analyzed using FlowJo software (V10.6.1), live cell and doublet discrimination was performed using FSC-A and SSC-A/H parameters, and FMO controls were used for gating. For the CD4-Tet staining, additional controls used for gating were the Human CLIP peptide control and uninfected animals. For the CD8-Tet staining, uninfected animal controls were used in addition to the FMO control to ensure proper gating.

### Statistical analyses

Statistical analyses were performed using Prism v.5.0 (GraphPad Software). When comparing more than 2 groups, one-way analysis of variance (ANOVA) was conducted followed by a Dunnett’s multiple comparison posttest to compare to the VC/S control. Comparisons between the infected groups were conducted using an unpaired student’s t-test. Statistical significance is designated by */# = p < 0.05, **/## = p < 0.01 and ***/### = p < 0.001.

## Results

### Triclosan does not augment influenza-induced morbidity

**Fig 2:**
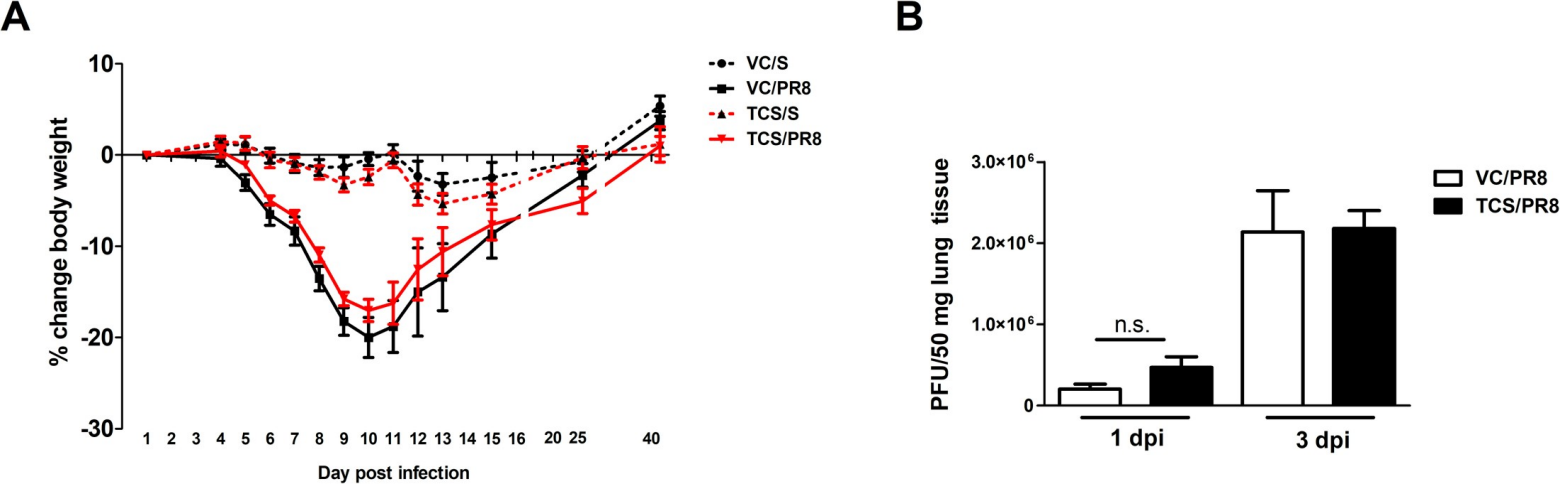
Morbidity and viral titers are unaltered by TCS. Mice treated with VC or TCS were intranasally infected with 50 pfu of PR8 or mock infected with S, as outlined in Fig 1. A) Mice were weighed throughout the course of infection and percent weight change was calculated based on starting weights n = 10 mice/group (through day 10 and 5 mice/group thereafter). B) Viral titers were assessed via plaque assays of lung tissue and calculated as plaques per 50 mg of lung tissue. N = 5 mice per group. No changes in body weights or viral titers due to TCS exposure were detected.

Mice that were exposed to triclosan (TCS) or acetone (VC) prior to being infected with PR8 or mock infected with S as outlined in Fig 1 were assessed for morbidity by tracking weights throughout the course of the study. Exposure to TCS alone did not result in weight loss as compared to the VC in the uninfected mice. All influenza infected mice lost a significant percentage of body weight, but there was no difference between the TCS and VC topically exposed mice. Additionally, weight loss peaked at a similar time between infected groups (10 dpi), and mice regained weight at a similar rate, indicating that TCS did not cause morbidity on its own or increase influenza associated morbidity (Fig 2A). As TCS can be absorbed by the body following dermal exposure [35, 36], we wanted to assess if TCS could directly or indirectly impact viral loads or replication. To this end, we assessed viral titers at 1 and 3 dpi by plaque assay and found no difference in viral loads between the VC/PR8 and TCS/PR8 groups (Fig 2B). Additionally, we did not find that dermal TCS exposure altered the kinetics of viral expansion and/or clearance (S2 Fig).

### Gene expression and protein analysis of lung and BALF

**Fig 3:**
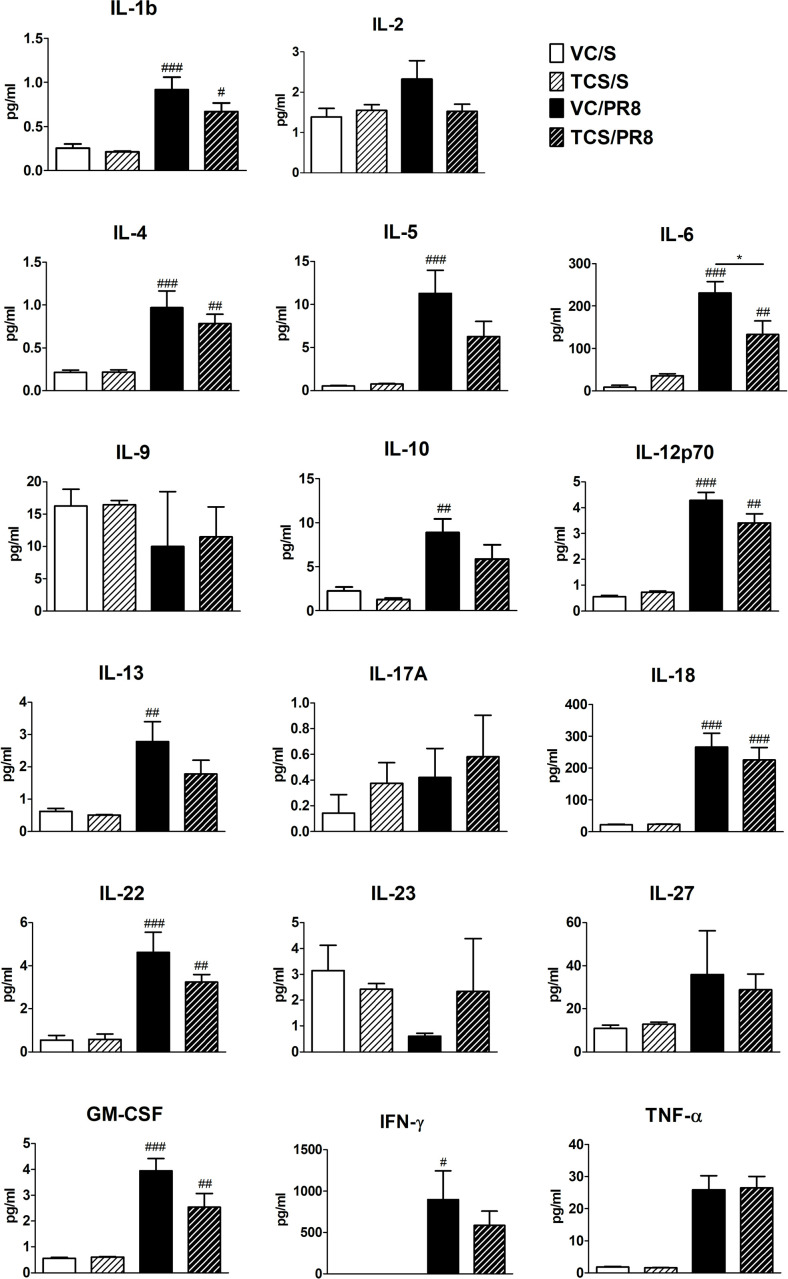
Cytokine expression in BALF. Cytokine expression was measured by Luminex assay in the first fraction of BALF isolated from mice at 10 dpi for each of the treatment groups, VC/S (white bars), TCS/S (light striped bars), VC/PR8 (black bars), and TCS/PR8 (dark striped bars). #s indicate significance as compared the VC/S control as determined by one-way ANOVA followed by a Dunnett’s post-test. *s indicate significance values determined using an unpaired student’s t-test between the VC/PR8 and TCS/PR8 groups. #/* = P <0.05, ## = p < 0.01, ### = p <0.001; n = 5 mice per group.

To begin to assess immune responses we performed gene expression and protein analysis in lung tissue and BALF, respectively, in all experimental conditions. As expected, genes associated with anti-viral Th1 responses were upregulated following influenza infection (*Ifng*, *Tbet*) and genes associated with Th2 responses were downregulated (*Il4*) (S3 Fig). No changes in *Gata3*, *Tnf*, *Ifnar1*, or *Tslp* were seen in the lung at this timepoint (S3 Fig). Interestingly, there were slightreductions in genes that are indicative of T cell migration (*Cxcr3;* p = 0.07) and T cell function (*Foxp3;* p = 0.08) in infected mice that were exposed to TCS (S3 Fig); these changes did not achieve statistical significance (p < 0.05). Cytokines in the BALF followed a similar pattern, with upregulation of inflammatory and T cell-associated cytokines seen in the PR8 infected mice (Fig 3). Some of these cytokines (IL-5, IL-13, IFN-γ and IL-10) were significantly upregulated only in the VC/PR8 group and not the TCS/PR8 group. While there was an overall trend in reduced expression of a number of cytokines in TCS exposed infected mice compared the VC exposed infected mine, a significant decrease in only BALF IL-6 was observed in the TCS/PR8 mice compared to the VC/PR8 mice (230.9 ± 26.67 vs 133.3 ± 31.52 pg/ml). Together, these data suggest that exposure to TCS might impact T cell responses to influenza infection.

### Triclosan reduces T cell responses to influenza infection

**Fig 4:**
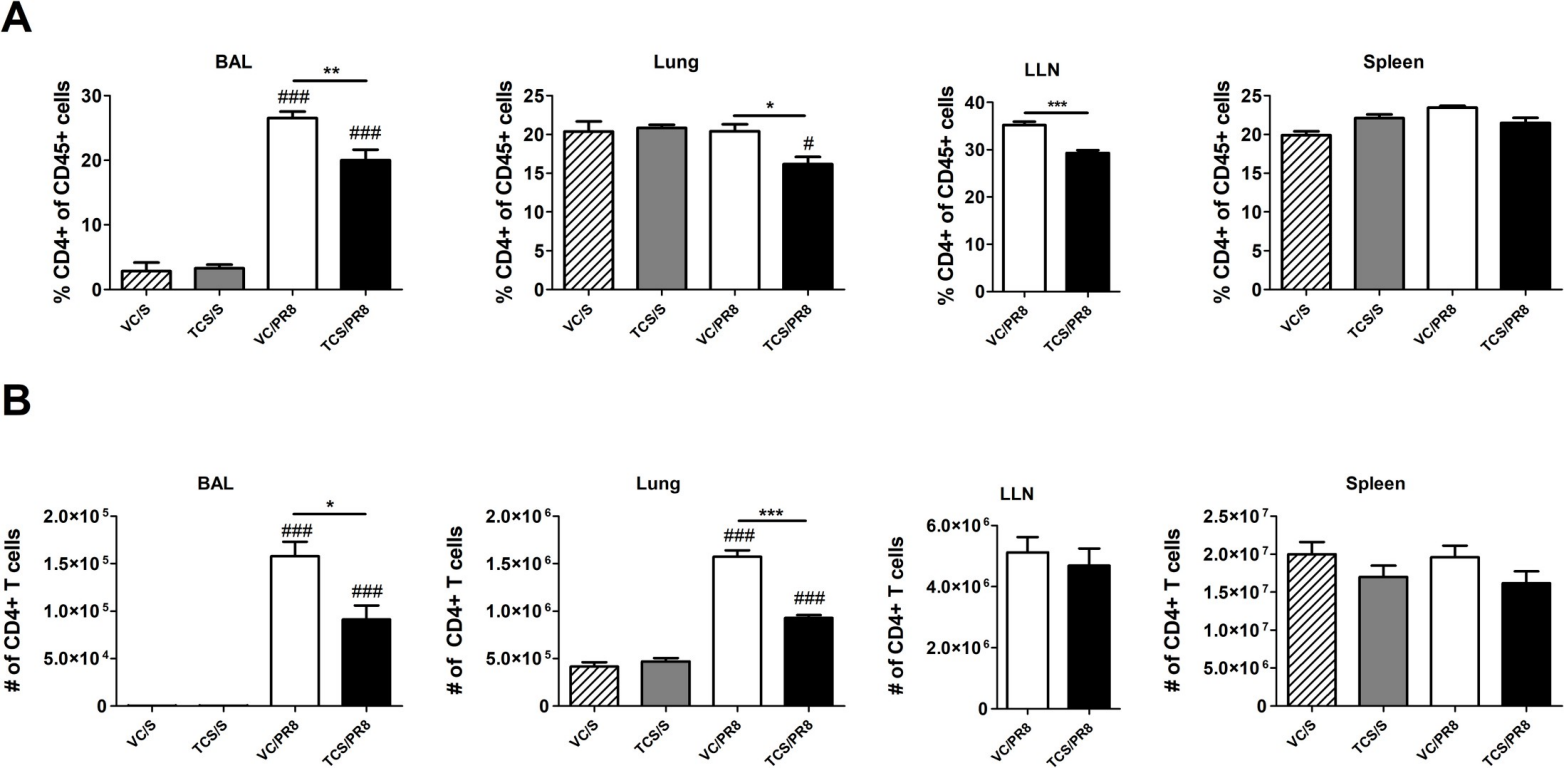
TCS reduces CD4 T cell responses in influenza infected mice. Total CD4 T cell frequencies (A) and numbers (B) were assessed in the BAL, lung, LLNs, and spleen, as indicated by flow cytometry. CD4 T cells were determined by gating on single cells followed by CD45+ cells, lymphocytes determined by FSC-A and SSC-A parameters, followed by CD4+ CD8- cells. Percent of CD4 T cells is graphed as a portion of total CD45+ cells. *s indicate significance between the VC/S and VC/PR8 groups as determined by unpaired student’s t-test. #s indicate significance as compared the VC/S control as determined by one-way ANOVA followed by a Dunnett’s post-test. * = P <0.05, ** = p < 0.01, *** = p <0.001; n = 5 mice per group.

**Fig 5:**
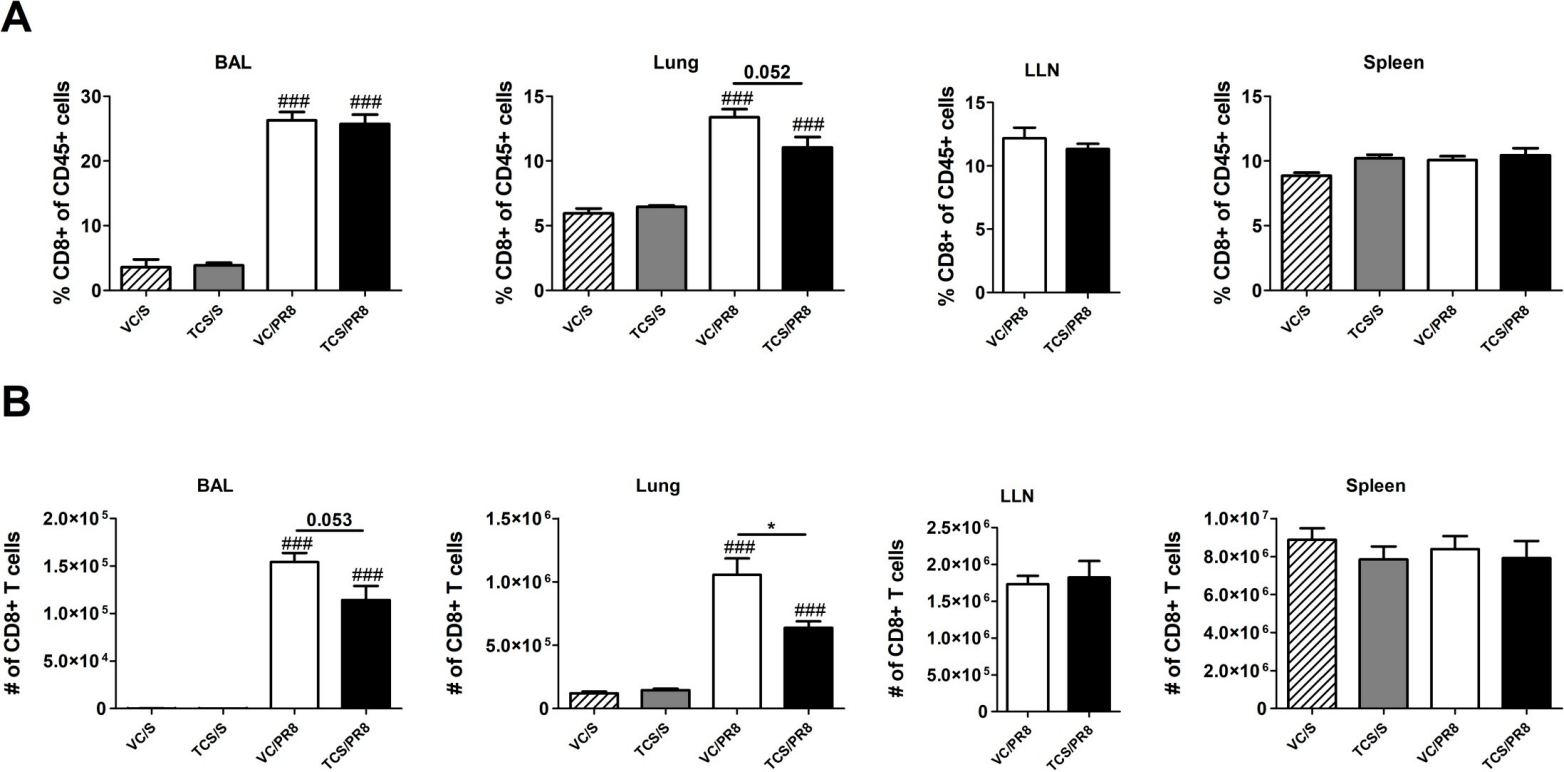
TCS reduces CD8 T cell responses in influenza infected mice. Total CD8 T cell frequencies (A) and numbers (B) were assessed in the BAL, lung, LLNs and spleen, as indicated by flow cytometry. CD8 T cells were determined by gating on single cells followed by CD45+ cells, lymphocytes determined by FSC-A and SSC-A parameters, followed by CD8+ CD4- cells. Percent of CD8 T cells is graphed as a portion of total CD45+ cells. *s indicate significance between the VC/S and VC/PR8 groups as determined by unpaired student’s t-test. # indicate significance as compared the VC/S control as determined by one-way ANOVA followed by a Dunnett’s post-test. * = P <0.05, ** = p < 0.01, *** = p <0.001; n = 5 mice per group.

Previous data from our laboratory and others has shown that TCS can skew T cell responses towards a Th2 phenotype [16]. As a strong Th1 T cell response is an integral part of the anti-influenza immune response, we assessed CD4+ and CD8+ T cells in the BAL, lung, LLNs, and spleen. No notable changes occurred at 40 dpi and the data within this manuscript focuses on results occurring at 10 dpi. Influenza infection increased the frequency of total CD4+ T cells in the BAL compared to mock infected controls (Fig 4A). However, in the TCS exposed mice the frequency of CD4+ T cells was reduced at the site of infection and proximal to the site of infection, with significant reductions seen in the BAL, lung, and LLNs compared to the VC/PR8 mice (Fig 4A). Numerically, influenza infection significantly increased the number of CD4+ T cell in the BAL and lung (Fig 4B). Due to constraints on cell numbers flow cytometry was only performed on LLNs collected from infected mice, however total cells increased in the LLNs of PR8 infected mice approximately 7-fold compared to mock-infected mice (1.38 × 10^7^ vs 1.89 × 10^6^, respectively). Reductions in the number of CD4+ T cells were observed in the TCS/PR8 infected mice as compared to the VC/PR8 infected mice in the BAL and the lung. Similarly, the frequency of CD8+ T cells in the BAL and lung was increased with influenza infection, and a reduction in total % of CD8+ T cells was seen in the lung of the TCS/PR8 mice compared to the VC/PR8 time (Fig 5A). Numerically, influenza infection increased CD8+ T cells at the site of infection (BAL and lung) and a reduction in number with TCS exposure was also observed at these sites (Fig 5B).

**Fig 6:**
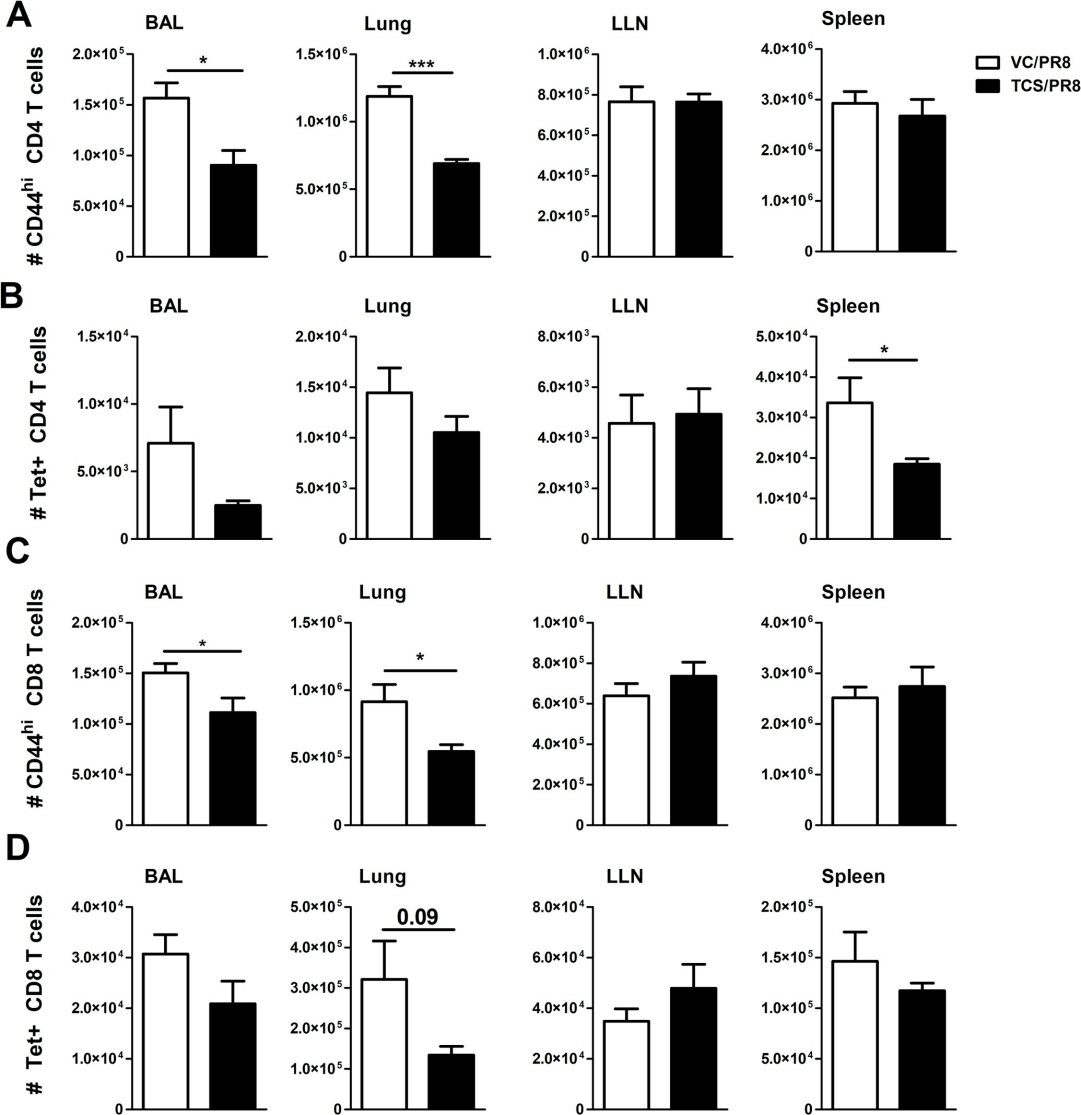
TCS reduces influenza-responding T cells. Influenza responding T cells were assessed in the indicated tissues in the VC/PR8 (white bars) and TCS/PR8 (black bars) groups. A) total number of CD44hi CD4 T cells B) total number of MHCII Tet+ CD4 T cells C) Total number of CD44hi CD8 T cells D) Total number of MHC I Tet+ CD8 T Cells. All populations analyzed were first gated on single cells, followed by CD45+ lymphocytes. Gating for CD44hi and Tet+ cells was determined using uninfected and FMO controls. N = 5 mice/ group. Significance between groups was determined using an unpaired student’s t-test. * = p < 0.05, ** = p < 0.01, *** = p <0.001; n = 5 mice per group.

We next assessed influenza-responding T cells by analyzing activated (CD44^hi^) CD4+ and CD8+ T cells, as well as influenza specific T cells by using MHCII and MHCI tetramers containing influenza A specific peptides to assess CD4+ and CD8+ T cell responses, respectively (Tet+ cells; S4 Fig). As expected, infection with influenza resulted in the increase in CD44^hi^ CD4+ and CD8+ T cells in all tissues assessed, except for the spleen, as compared to the mock infected animals. Furthermore, there was no difference in frequency or number of CD44^hi^ cells between the mice exposed to acetone (VC/S) and those exposed to TCS within the mock infected groups (S1 Table). However, when comparing the mice that were infected with influenza (VC/PR8 *vs*. TCS/PR8) there was a reduction in the total number of CD44^hi^ CD4+ T cells in the lung and BAL of the TCS exposed mice (Fig 6A). Similarly, a significant reduction in the number of CD44^hi^ CD8+ T cells was seen in the BAL and the lung for the TCS exposed group compared to the VC (Fig 6C). A significant reduction in Tet+CD4+ T cells was seen in the spleen (Fig 6B), however reductions in influenza specific CD4 and CD8 T cells in the lung and lung airways (BAL) did not achieve statistical significance (Fig 6B and 6D).

### Exposure to triclosan reduces T-bet expression in influenza responding T cells

**Fig 7:**
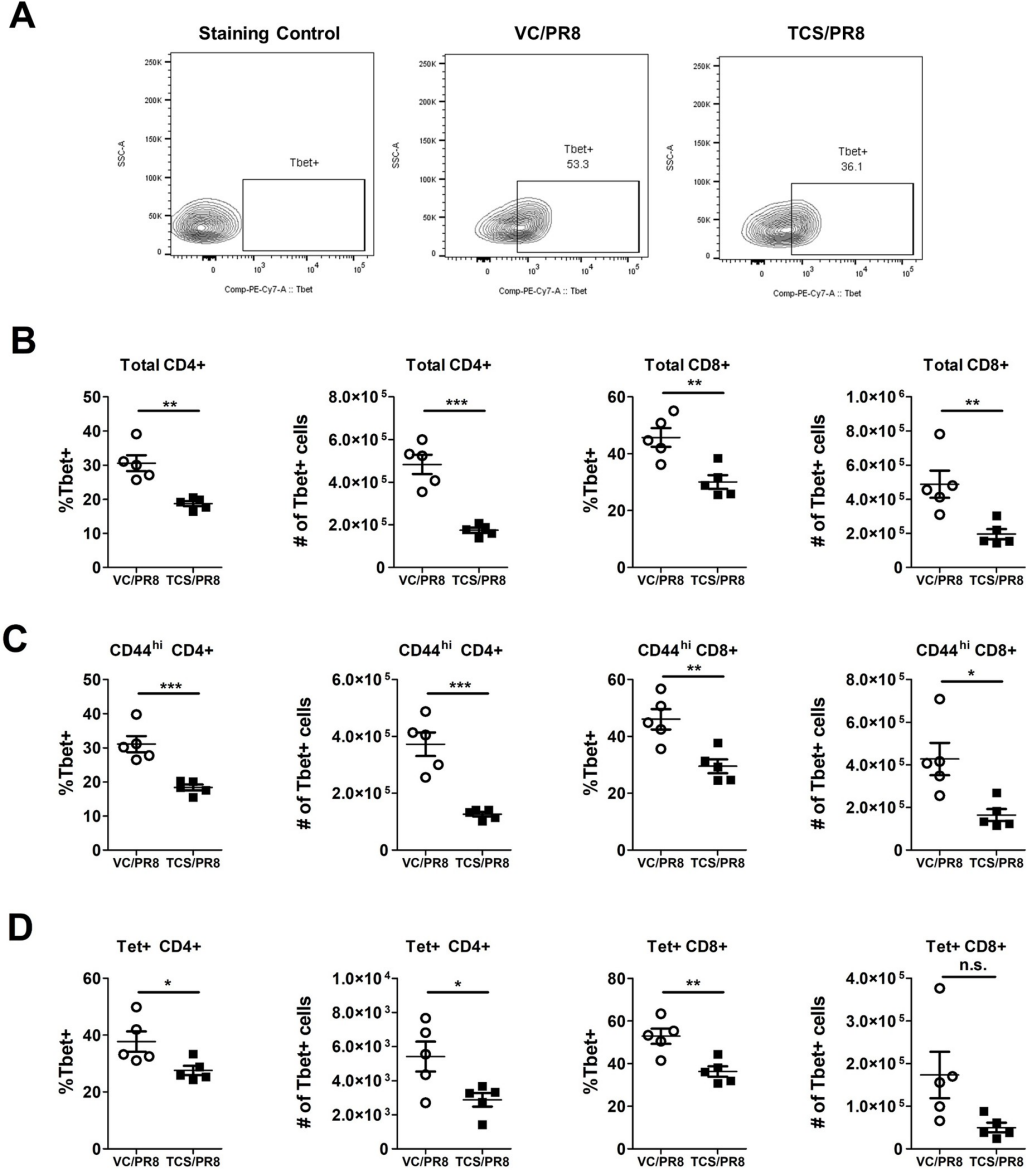
T-bet expression is reduced in influenza infected mice exposed to TCS. Influenza responding T cells were assessed in the indicated tissues in the VC/PR8 (white circles) and TCS/PR8 (black squares) groups for expression of T-bet via flow cytometry. A) Representative gating for T-bet expression shown with an FMO control, VC/PR8 mouse and TCS/PR8 mouse, from lung tissue. Cells were previously gated on, single cells, CD45+ lymphocytes, Tet+ CD8+ T cells. B) Frequencies and numbers of lung T-bet+ cells of total CD4+ and CD8+ T cells, C) Frequencies and numbers of lung T-bet+ cells of CD44^hi^ CD4+ and CD8+ T cells D) Frequencies and numbers of lung T-bet+ cells of Tet+ CD4+ and CD8+ T cells. N = 5 mice/ group. Significance between groups was determined using an unpaired student’s t-test. * = p < 0.05, ** = p < 0.01, *** = p <0.001; n = 5 mice per group.

**Fig 8:**
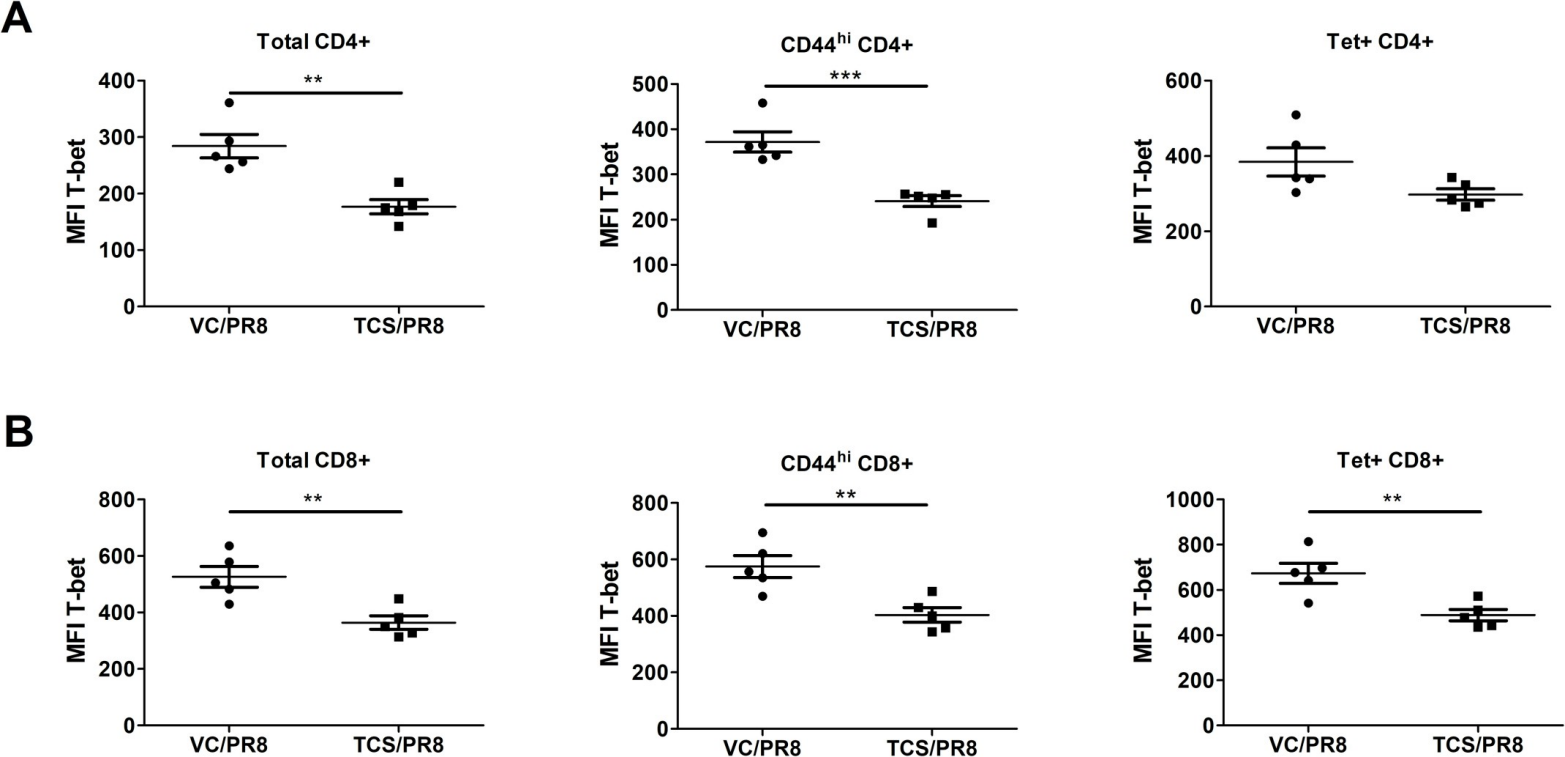
T-bet expression is reduced on a per-cell basis on lung T cells responding to influenza infection in TCS exposed mice. Median fluorescence intensity (MFI) of T-bet expression was measured on total, activated (CD44^hi^), and Tet+ CD4+ (A) and CD8+ (B) T cells isolated from the lungs of mice infected with PR8 at 10 dpi. *s indicate significance values determined using an unpaired student’s t-test between the VC/PR8 and TCS/PR8 groups. ** = p < 0.01, *** = p <0.001; n = 5 mice per group.

To determine if exposure to TCS might affect the functional capabilities of influenza responding T cells, we analyzed the expression of the canonical Th1 and Th2 transcription factors, T-bet and GATA3, respectively. Overall, the expression of GATA3 was low/not expressed in T cells isolated from the assessed sites and was unchanged by TCS exposure (S2 Table) Mirroring the gene expression data, influenza infection resulted in the upregulation of T-bet expression (MFI and frequency/number of T-bet+ cells) in CD4+ and CD8+ T cells compared to uninfected controls (S1 Table). Within the infected groups, T-bet expression was unchanged in the LLNs, minor changes in the number of T-bet+ cells was seen in the BAL with significant reductions in the number of T-bet+CD44^hi^CD4+ T cells, and significant differences in the frequency and number of T-bet+ CD44^hi^CD8+ T cells and Tet+CD8+ T cells were observed in the spleen (S3 Table). The most dramatic changes in T-bet expression were in the lung cells (Fig 7A) of PR8 infected mice. In cells isolated from the lung tissue, TCS exposure resulted in a significant reduction in the frequency and number of T-bet+ total CD4+ and CD8+ T cells (Fig 7B), activated (CD44^hi^) CD4+ and CD8+ T cells (Fig 7C), as well as influenza specific Tet+ CD4+ and CD8+ T cells, in both frequency and number (Fig 7D). In addition to a decreased frequency/number of T-bet+ lung CD4+ and CD8+ T cells responding to influenza infection in TCS treated mice, total (CD4+ and CD8+), CD44^hi^ (CD4+ and CD8+), and Tet+ (CD8+) T cells in the lung had a decreased level of T-bet expression on a per cell basis (decreased T-bet MFI; Fig 8A and 8B).

## Discussion

It is widely recommended to use antimicrobial agents for disinfection and sterilization in hospitals and healthcare facilities to prevent transmission of infectious pathogens among workers and patients. However, at the same time, we must recognize the potential hazards associated with dermal exposure to these antimicrobial chemicals and take steps to mitigate their negative health effects, if possible. In order to do this, we need to be aware of all potential health effects that can occur due to exposure. The majority of studies focus on the direct effect of chemical antimicrobials, whether the chemical is toxic or allergenic itself. Several studies have investigated secondary (indirect) effects of dermal exposure to antimicrobial chemicals, including the augmentation of Th2 responses [16], and alterations of the commensal microbiota of the skin [37, 38], all which might contribute to alterations of subsequent immune responses [39]. Here, we show that TCS alters the immune response to influenza virus infection in a murine model.

The concept that the immune response to one pathogen can change the immune response to a subsequent pathogen has been well studied in the field of infectious diseases. Although co-exposure studies are complex, and there are almost always exceptions to the rule, in general, infections that result in a strong Th2 bias negatively impact a host’s ability to respond appropriately to a Th1 stimulus. This has been largely studied in relation to parasitic helminth infections, as these infections are common, persistent in nature, and are known to invoke strong Th2 polarizing responses [30]. Impaired T cell responses have been seen in response to Vaccinia Virus infections in mice that are co-infected with *Ascaris* [40] and *Schistosoma mansoni* [41], with indications in human populations showing that responses to vaccinations (Th1 reliant) are impaired in helminth infected individuals [42]. While the Th2 polarizing effects of chemicals are less well studied than those of helminth infections, this study shows that effects similar to those observed in these parasitic models can be elicited with application of the chemical antimicrobial TCS, particularly in regard to reductions in T cell responses. While we did not see increases in GATA3 expression in the TCS exposed mice in the lung or LLNs, we know from previous studies in our lab that TCS results in increases in GATA3 expression in LNs draining the site of chemical application [16]. Without a stimulus in the lung (mock infected mice) T cells responding to TCS (Th2 skewed) are unlikely to traffic to the lung, and changes in GATA3 at this distal site would not be anticipated. In the infected mice, it is possible that the overwhelming Th1 response induced by influenza infection in the mouse lung reduced GATA3 levels below the level of detection as T-bet can directly inhibit GATA3 expression [43]. Despite the lack of induction of Th2 responses in the lung environment, TCS still alters the immune response to influenza, highlighting the systemic and interconnected nature of immune responses.

In this manuscript we show that exposure to TCS resulted in the reduction of total CD4+ and CD8+ T cell responses (Figs 4 and 5) as well as activated (CD44+) T cells responding to infection (Fig 6) at the peak of the T cell response (10 dpi). While the defects in T cell responses in this study were not drastic, the numerical defects observed in T cell responses at the site of infection were consistent, occurring in both the BAL and the lung, and found in both CD4+ and CD8+ T cell subsets. Overall, more significant numerical changes were seen when looking at total and activated (CD44^hi^) CD4+ and CD8+ T cells compared to the Tet+ T cells. The MHC I/II tetramers used here to detect influenza-specific T cells that are responding to specific influenza epitopes HA_143-155_ and NP_147-155_, and therefore only capture a portion of the total anti-influenza T cell response, while the CD44^hi^ population accounts for all activated T cells in the specified tissue. Under resting conditions T cells are not typically found in the BAL; as seen in Figs 4 and 5, negligible numbers of CD4+ and CD8+ T cells are seen in non-infected groups. As such, it can be assumed that the majority of T cells and especially CD44^hi^ cells found in the BAL are responding to the infection and were decreased with TCS exposure. Future studies should include analysis of alternative and subdominant epitopes to obtain a more complete picture of TCS’s effect on the total anti-influenza immune response.

In addition to the defects in T cell responses we also found a small, but consistent, reduction in the levels of cytokines involved with T cell responses in the BAL fluid in the TCS/PR8 group compared to the VC/PR8 group (Fig 3). The differences in IL-6 expression reached statistical significance. IL-6 is produced in response to tissue damage and infections by a variety of cell types including fibroblasts, keratinocytes, endothelial cells, mast cells, antigen presenting cells and T and B cells [44]. Importantly, IL-6 has been shown to contribute to T cell responses in influenza infection, and mice deficient in IL-6 show reduced T cell numbers in the lung and LLNs [45]. As the changes in the remainder of cytokines produced by T cells did not reach statistical significance and occurred in both Th1 (IFN-γ, IL-12P70) and Th2 (IL-4, IL-5, and IL-13) associated cytokines, we believe this is likely reflective of the numerical reduction in infiltrating leukocytes including CD4+ and CD8+ T cells rather than a functional defect in the Th1 cell population.

One of the particularly compelling pieces of data in this study was the reduction in T-bet expression seen in the responding CD4+ and CD8+ T cells in the lung of mice exposed to TCS (Figs 7 and 8). T-bet serves as the master transcription factor for Th1 immune responses [46] and is known to be important for the clearance of several intracellular pathogens. Despite the reduction in T-bet+ T cells in the lung, in our studies we did not see a decrease in viral clearance (Figs 2B and S2). Fitting with this finding, a recent paper by Dhume and colleagues [47] showed that the protective efficacy of T-bet deficient CD4+ T cells responding to PR8 is only marginally reduced. In these studies, the reduced efficacy was due to lower CXCR3 expression on the CD4+ T cells, which led to reduced accumulation of these cells in the influenza infected lung [47]. Similarly, in our studies, along with reduced T-bet expression we found reduced *Cxcr3* expression (p = 0.07; S3 Fig) in the lung of TCS exposed mice when comparing the infected groups. As CXCR3 is a known target of T-bet, and is important for CD4+ T cell trafficking to sites of inflammation [48], this provides insight into a potential mechanism behind the reduced frequency and numbers of T cells found at, and adjacent to, the site of infection we found in our studies. If the defect is based on the ability of cells to migrate to the site of infection, it might also explain why numbers were reduced in the BAL and the lung, with no numerical defect found in the LLNs (Figs 4–6). T-bet’s effect on CXCR3 expression on CD8+ T cells is less clear, with studies correlating T-bet expression with both increased and decreased CXCR3 expression on this cell population [49]. Interestingly, IL-6 has also been tied to T cell migration into sites of inflammation, with mice deficient in IL-6 expressing a lower level of a number of chemokine receptors including CXCR3 [50]. Further studies investigating the migratory ability of T cells following TCS exposure might provide more insight regarding the mechanisms behind reduced T cell responses to influenza infection in this model.

As previously mentioned, despite the reduction in T cells and T-bet expression in T cells found in the lung, there were no changes observed in viral loads or in morbidity of the infected mice that were treated with TCS (Fig 2). This result is not surprising, as the immune systems is equipped with multiple compensatory and redundant mechanisms for protection against pathogens. Natural killer cells, which were not assessed in this study, play an important role in early viral control and pathogenicity, with many functions redundant to T cell responses in the lung including cytotoxicity and cytokine production [51]. Due to the integrated roles that many of these cells play the loss of multiple cell subsets if often necessary to see an overwhelming effect on viral clearance. Notably, influenza virus can be cleared from the lungs of mice in the absence of CD8 T cells, or CD4 T cells, but cannot be cleared in the absence of both compartments [28], highlighting the ability of the immune system to compensate in the absence of a cell type.

While these studies were conducted with the antimicrobial TCS, other chemical antimicrobials have been shown to promote Th2 responses [52–55] and can augment allergic responses [3]; it would not be a stretch to believe that other chemicals with similar immunological properties could also impact anti-viral immune responses. Additionally, as healthcare workers are likely exposed to multiple antimicrobial chemicals at a time, and for extended periods of time, the cumulative effects of these chemicals might result cause immunomodulatory effects. We have shown that extended exposures increase Th2 immune responses [54], and that chemical co-exposures can increase markers of allergic disease including IL-4 levels and total IgE [56]. It is possible an extended duration of TCS exposure might lead to an enhanced impact on T cell responses. Additionally, most intracellular pathogens, including other respiratory viruses, are dependent on the development of Th1 immune responses, therefore it is quite possible that the results obtained in this study are also relevant to the development of immune responses to other respiratory viral pathogens, although to confirm this, additional studies would need to be conducted.

Overall, the results obtained from this study show that dermal exposure to the antimicrobial TCS results in a reduction in the number of T cells responding to influenza infection in BALB/c mice. These changes likely occur due, in part, to a reduced expression of T-bet that is seen in the infected TCS exposed mice compared to the infected controls. While in this study we did not see increases in morbidity or mortality to influenza due to TCS exposure, the reductions in T cell responses indicate an altered immune response which might impact proper development of recall responses upon subsequent exposure. The implications of these results are that individuals, such as healthcare workers, who are exposed to high levels and prolonged use of immune-altering chemical antimicrobials may have sub-optimal responses to respiratory viral infections, making them more susceptible to disease and re-infection. Based on these results, more studies are warranted to determine if this is a common attribute of antimicrobials, and what are the implications for healthcare workers.

## Supporting information

S1 Fig Range finding study for PR8 dosage used in study. Mice were infected with 5–1000 pfu of PR8, or saline control as indicated in the figure legend. Mice were monitored daily following infection in order to determine the evident lethal dose as defined by a body weight loss of greater than 20% and signs of morbidity including pulmonary distress. Mice exhibiting these symptoms were humanely euthanized. Percent survival (A) and average percent change in body weight (B) was determined for each group, and 50 pfu (pink line) was chosen as the sub-lethal infectious dose to be used for subsequent studies.
(DOCX)

S2 Fig Viral load kinetics in PR8 infected mice exposed to TCS or VC. Mice were infected with 500 pfu of PR8, and exposed to VC (black line) or TCS (red line) as outlined in Fig 1. Lungs were collected for assessment of viral titers using a combined TCID50/HAI assay. Results are shown as TCID50/ml. n = 5–3 mice per group; no significant differences between treatment groups were found.
(DOCX)

S3 Fig Gene expression analysis of T cell associated genes in the lung. An aliquot of cells obtained from the single cell suspension of lung tissue was used for the assessment of gene expression at 10 dpi. Relative fold gene expression changes (2^-ΔΔCT^) were determined compared to the VC/S control and normalized for expression of housekeeping gene *Actb*. #s indicate significance as compared the VC/S control as determined by one-way ANOVA followed by a Dunnett’s post-test. p values between the VC/PR8 and TCS/PR8 groups were determined using an unpaired student’s t-test. # = P <0.05, ## = p < 0.01, ### = p <0.001; n = 5 mice per group.
(DOCX)

S4 Fig Representative tetramer staining. Representative staining for the influenza A HA_143-155_ MHC II (I-A(d)/ HNTNGVTAACSHE) tetramer (top) and influenza A NP_147-155_ MHC I (H-2K(d) /TYQRTRALV) tetramer (bottom) to detect influenza specific CD4+ and CD8+ T cells, respectively. For the irrelevant peptide control sample (human CLIP peptide MHC II (I-A(d)/ PVSKMRMATPLLMQA) tetramer; top left) a portion of cells were pooled from all infected mice (from both the VC and TCS groups) and an aliquot of that sample was stained. Staining shown for the influenza specific tetramers are from a single mouse each, but are representative of the other samples. Cells were previously gated on single cells, CD45+ cells, lymphocytes, and CD4+ (top) or CD8+ (bottom) cells.
(DOCX)

S1 Table CD44 and T-bet expression in mock infected animals. Significance between groups was assessed using an unpaired student’s t-test; no significant changes were found.
(DOCX)

S2 Table Frequency of GATA3+ T cells. Significance was assessed between all groups compared the VC/S control as determined by one-way ANOVA followed by a Dunnett’s post-test and by using an unpaired student’s t-test between the VC/PR8 and TCS/PR8 groups; no significant changes were found. $- not enough cells to accurately assess level of GATA3 expression.
(DOCX)

S3 Table T-bet expression in activated and influenza responding T cells. *s indicate significance against VC/PR8 control determined by an unpaired student’s t-test. * = P <0.05, ** = p < 0.01; n = 5 mice per group.
(DOCX)
