# Supplementary material for: Topical exposure to triclosan inhibits Th1 immune responses and reduces T cells responding to influenza infection in mice
Source: PLoS One. 2020 Dec 29;15(12):e0244436. doi: 10.1371/journal.pone.0244436 (PMC7771851; doi:10.1371/journal.pone.0244436)
Supplement: S1 Fig — Mice were infected with 5–1000 pfu of PR8, or saline control as indicated in the figure legend. Mice were monitored daily following infection in order to determine the evident lethal dose as defined by a body weight loss of greater than 20% and signs of morbidity including pulmonary distress. Mice exhibiting these symptoms were humanely euthanized. Percent survival (A) and average percent change in body weight (B) was determined for each group, and 50 pfu (pink line) was chosen as the sub-lethal infectious dose to be used for subsequent studies. (DOCX) [file pone.0244436.s001.docx]

**
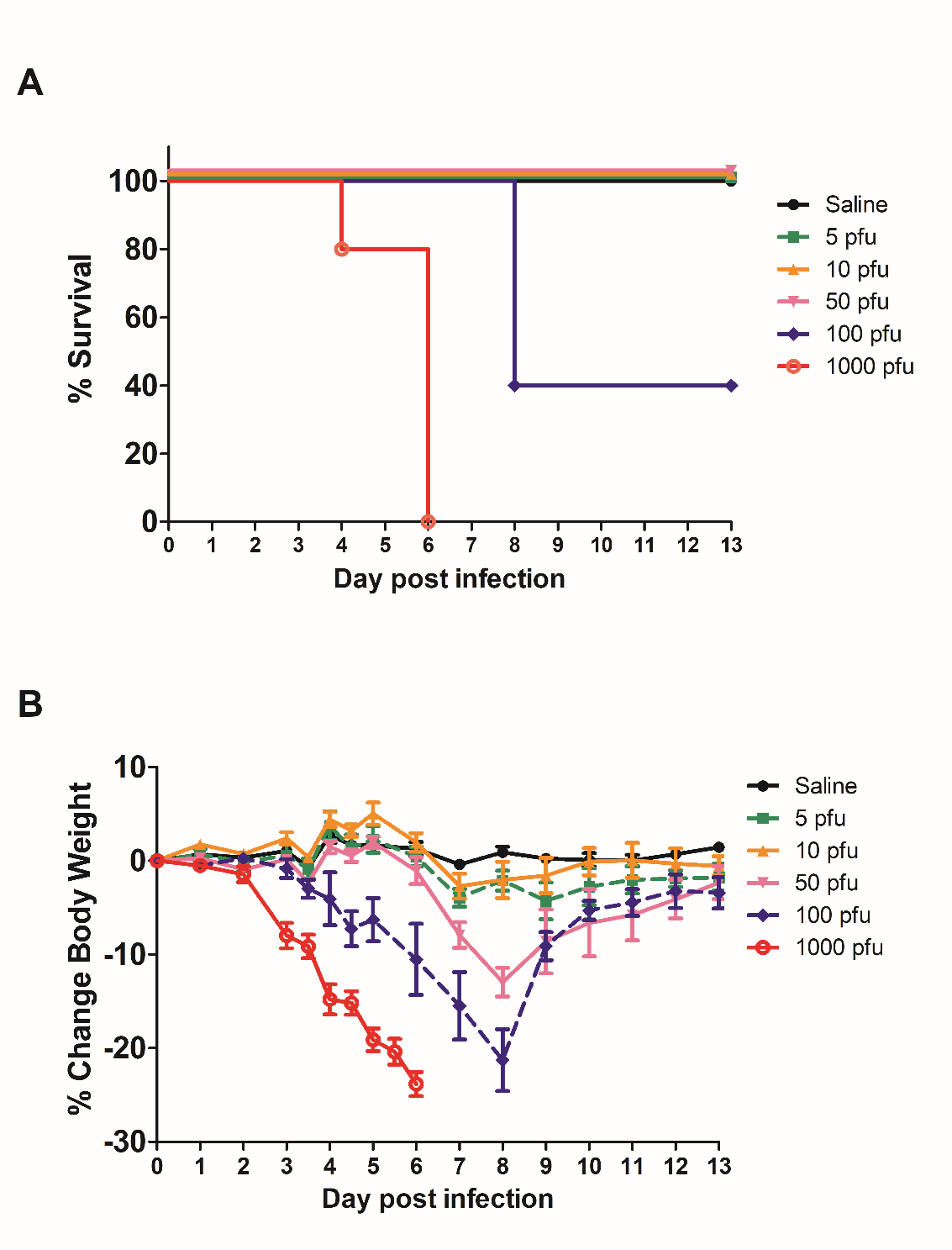
**

**S1 Fig. Range finding study for PR8 dosage used in study.** Mice were infected with 5-1000 pfu of PR8, or saline control as indicated in the figure legend. Mice were monitored daily following infection in order to determine the evident lethal dose as defined by a body weight loss of greater than 20% and signs of morbidity including pulmonary distress. Mice exhibiting these symptoms were humanely euthanized. Percent survival (A) and average percent change in body weight (B) was determined for each group, and 50 pfu (pink line) was chosen as the sub-lethal infectious dose to be used for subsequent studies.
