# Supplementary material for: Topical exposure to triclosan inhibits Th1 immune responses and reduces T cells responding to influenza infection in mice
Source: PLoS One. 2020 Dec 29;15(12):e0244436. doi: 10.1371/journal.pone.0244436 (PMC7771851; doi:10.1371/journal.pone.0244436)
Supplement: S2 Fig — Mice were infected with 500 pfu of PR8, and exposed to VC (black line) or TCS (red line) as outlined in Fig 1. Lungs were collected for assessment of viral titers using a combined TCID50/HAI assay. Results are shown as TCID50/ml. n = 5–3 mice per group; no significant differences between treatment groups were found. (DOCX) [file pone.0244436.s002.docx]

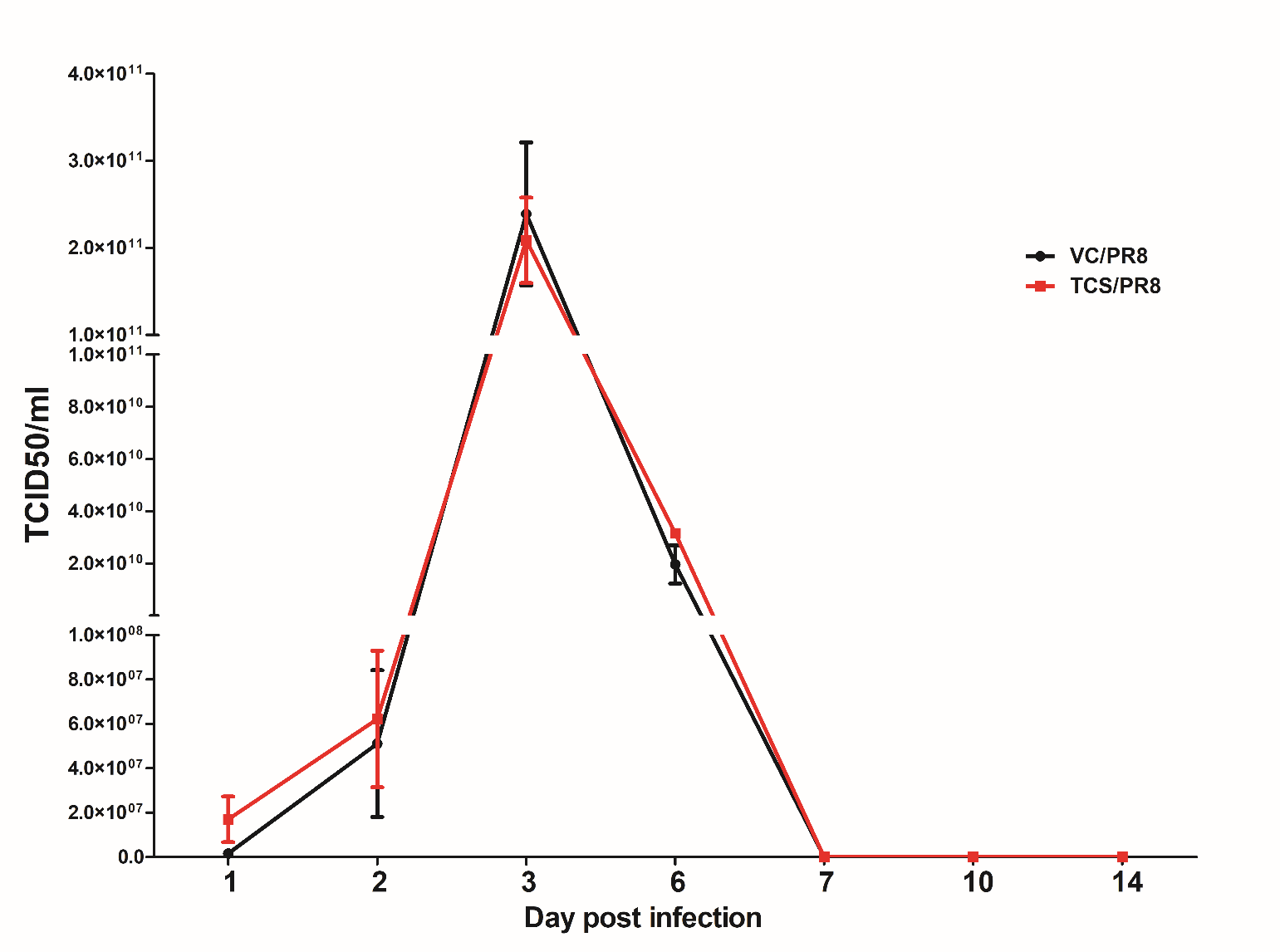


**S2 Fig. Viral load kinetics in PR8 infected mice exposed to TCS or VC.** Mice were infected with 500 pfu of PR8, and exposed to VC (black line) or TCS (red line) as outlined in Figure 1. Lungs were collected for assessment of viral titers using a combined TCID50/HAI assay. Results are shown as TCID50/ml. n= 5-3 mice per group; no significant differences between treatment groups were found.
