# Supplementary material for: Topical exposure to triclosan inhibits Th1 immune responses and reduces T cells responding to influenza infection in mice
Source: PLoS One. 2020 Dec 29;15(12):e0244436. doi: 10.1371/journal.pone.0244436 (PMC7771851; doi:10.1371/journal.pone.0244436)
Supplement: S3 Fig — An aliquot of cells obtained from the single cell suspension of lung tissue was used for the assessment of gene expression at 10 dpi. Relative fold gene expression changes (2-ΔΔCT) were determined compared to the VC/S control and normalized for expression of housekeeping gene Actb. #s indicate significance as compared the VC/S control as determined by one-way ANOVA followed by a Dunnett’s post-test. p values between the VC/PR8 and TCS/PR8 groups were determined using an unpaired student’s t-test. # = P <0.05, ## = p < 0.01, ### = p <0.001; n = 5 mice per group. (DOCX) [file pone.0244436.s003.docx]

**
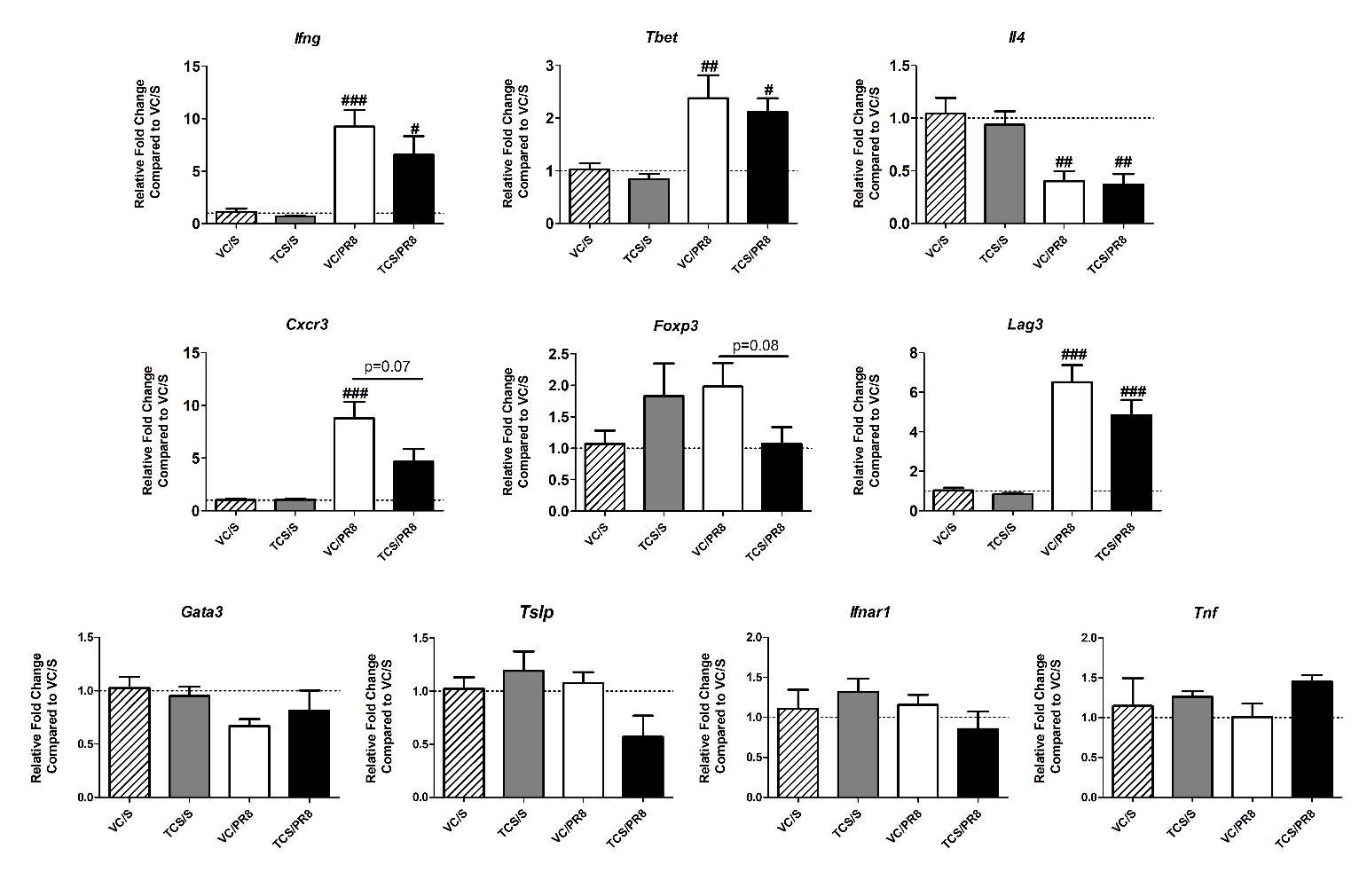
**

**S3 Fig. Gene expression analysis of T cell associated genes in the lung.** An aliquot of cells obtained from the single cell suspension of lung tissue was used for the assessment of gene expression at 10 dpi. Relative fold gene expression changes (2^-ΔΔCT^) were determined compared to the VC/S control and normalized for expression of housekeeping gene *Actb*. #s indicate significance as compared the VC/S control as determined by one-way ANOVA followed by a Dunnett’s post-test. p values between the VC/PR8 and TCS/PR8 groups were determined using an unpaired student’s t-test. # = P <0.05, ## = p < 0.01, ### = p <0.001; n= 5 mice per group.
