# Supplementary material for: Topical exposure to triclosan inhibits Th1 immune responses and reduces T cells responding to influenza infection in mice
Source: PLoS One. 2020 Dec 29;15(12):e0244436. doi: 10.1371/journal.pone.0244436 (PMC7771851; doi:10.1371/journal.pone.0244436)
Supplement: S4 Fig — Representative staining for the influenza A HA143-155 MHC II (I-A(d)/ HNTNGVTAACSHE) tetramer (top) and influenza A NP147-155 MHC I (H-2K(d) /TYQRTRALV) tetramer (bottom) to detect influenza specific CD4+ and CD8+ T cells, respectively. For the irrelevant peptide control sample (human CLIP peptide MHC II (I-A(d)/ PVSKMRMATPLLMQA) tetramer; top left) a portion of cells were pooled from all infected mice (from both the VC and TCS groups) and an aliquot of that sample was stained. Staining shown for the influenza specific tetramers are from a single mouse each, but are representative of the other samples. Cells were previously gated on single cells, CD45+ cells, lymphocytes, and CD4+ (top) or CD8+ (bottom) cells. (DOCX) [file pone.0244436.s004.docx]

**
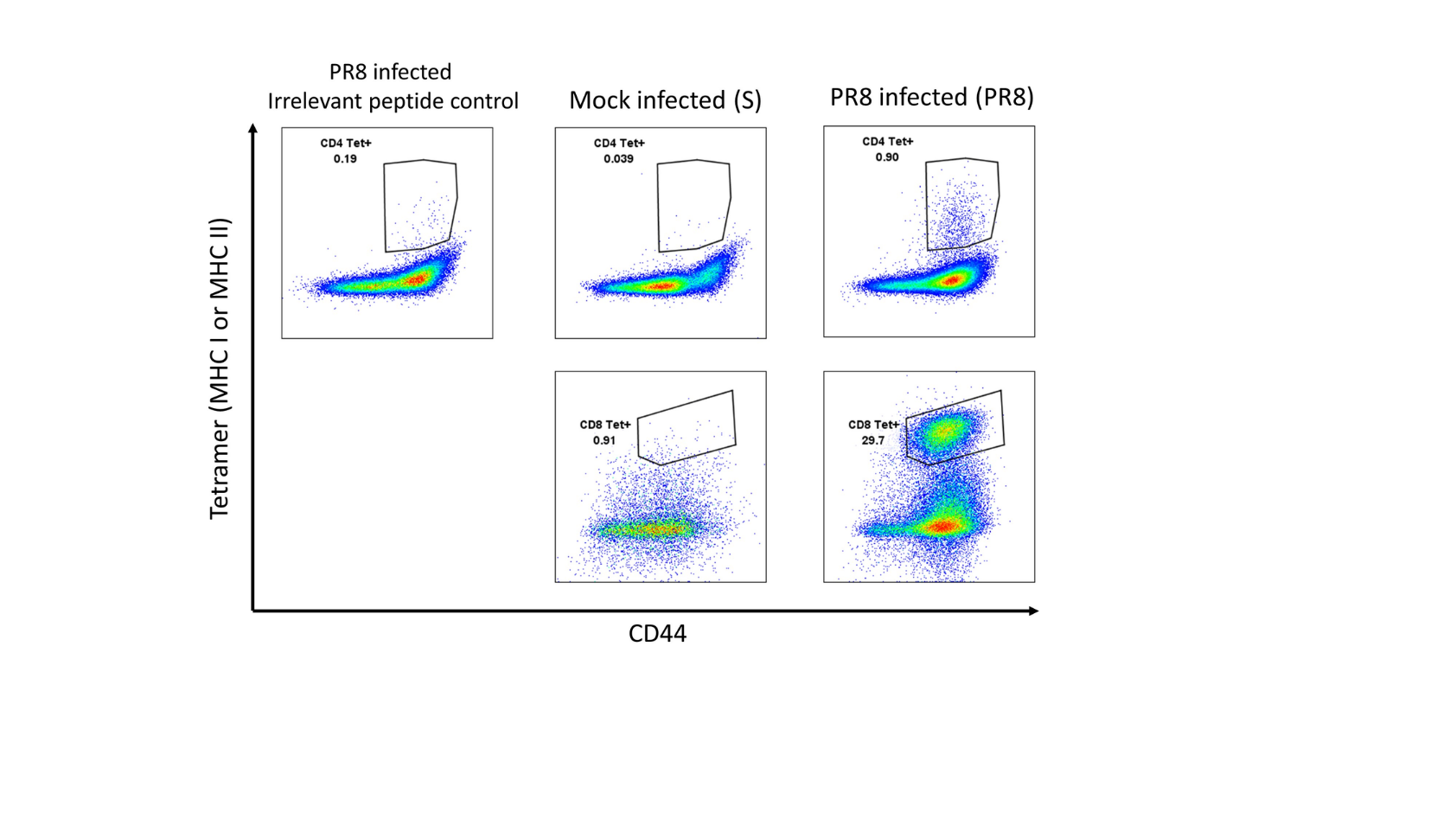
**

**S4 Fig. Representative tetramer staining.** Representative staining for the influenza A HA_143-155_ MHC II (I-A(d)/ HNTNGVTAACSHE) tetramer (top) and influenza A NP_147-155_ MHC I (H-2K(d) /TYQRTRALV) tetramer (bottom) to detect influenza specific CD4+ and CD8+ T cells, respectively. For the irrelevant peptide control sample (human CLIP peptide MHC II (I-A(d)/ PVSKMRMATPLLMQA) tetramer; top left) a portion of cells were pooled from all infected mice (from both the VC and TCS groups) and an aliquot of that sample was stained. Staining shown for the influenza specific tetramers are from a single mouse each, but are representative of the other samples. Cells were previously gated on single cells, CD45+ cells, lymphocytes, and CD4+ (top) or CD8+ (bottom) cells.
