# Supplementary material for: Topical exposure to triclosan inhibits Th1 immune responses and reduces T cells responding to influenza infection in mice
Source: PLoS One. 2020 Dec 29;15(12):e0244436. doi: 10.1371/journal.pone.0244436 (PMC7771851; doi:10.1371/journal.pone.0244436)
Supplement: S1 Table — Significance between groups was assessed using an unpaired student’s t-test; no significant changes were found. (DOCX) [file pone.0244436.s005.docx]

**S1 Table. CD44 and T-bet expression in mock infected animals**

|  | **BAL** | | **Lung** | | **Spleen** | |
| --- | --- | --- | --- | --- | --- | --- |
|  | VC/S | TCS/S | VC/S | TCS/S | VC/S | TCS/S |
| **CD44^hi^ CD4+ (%)** | 80.3 ± 12.71 | 78.48 ± 6.03 | 22.40 ± 1.73 | 21.78 ± 0.79 | 15.02 ± 1.16 | 15.26 ± 0.43 |
| **CD44^hi^ CD4+ (#)** | 203.1 ± 83.96 | 186.3 ± 37.38 | 90088 ± 5324 | 101594 ± 5914 | 3008000 ± 353415 | 2595000 ± 228357 |
| **CD44^hi^ CD8+ (%)** | 63.13 ± 8.80 | 46.76 ± 1.58 | 61.26 ± 1.24 | 60.98 ± 1.87 | 30.54 ± 1.43 | 32.62 ± 1.40 |
| **CD44^hi^ CD8+ (#)** | 212.0 ± 68.17 | 134.0 ± 24.06 | 74298 ± 9281 | 89343 ± 8450 | 2703000 ± 207743 | 2576000 ± 286235 |
| **T-bet+ CD4+ (%)** | 8.55 ± 3.22 | 8.93 ± 2.07 | 5.60 ± 0.34 | 5.11 ± 0.45 | 0.13 ± 0.01 | 0.17 ± 0.005 |
| **T-bet+ CD4+ (#)** | 29.24 ± 17.37 | 24.14 ± 9.61 | 22692 ± 1724 | 23364 ± 686.2 | 26322 ± 1040 | 28827 ± 3223 |
| **T-bet+ CD8+ (%)** | 18.72 ± 2.99 | 22.12 ± 2.68 | 7.85 ± 1.28 | 6.7 ± 1.54 | 0.11 ± 0.02 | 0.08 ± 0.02 |
| **T-bet+ CD8+ (#)** | 58.26 ± 17.35 | 60.07 ± 9.08 | 8925 ± 635.4 | 9260 ± 1472 | 9832 ± 2361 | 7060 ± 2633 |
| **MFI T-bet CD4s** | 9.52 ± 21.59 | 53.42 ± 22.76 | 24.21 ± 5.15 | 13.99 ± 6.40 | 37.74 ± 4.96 | 42.86 ± 4.31 |
| **MFI T-bet CD8s** | 146.1 ± 29.8 | 141.1 ± 25.75 | 41.98 ± 11.0 | 23.58 ± 11.75 | 34.68 ± 4.89 | 37.74 ± 2.93 |

Significance between groups was assessed using an unpaired student’s t-test; no significant changes were found.
