# Supplementary material for: Topical exposure to triclosan inhibits Th1 immune responses and reduces T cells responding to influenza infection in mice
Source: PLoS One. 2020 Dec 29;15(12):e0244436. doi: 10.1371/journal.pone.0244436 (PMC7771851; doi:10.1371/journal.pone.0244436)
Supplement: S2 Table — Significance was assessed between all groups compared the VC/S control as determined by one-way ANOVA followed by a Dunnett’s post-test and by using an unpaired student’s t-test between the VC/PR8 and TCS/PR8 groups; no significant changes were found. $- not enough cells to accurately assess level of GATA3 expression. (DOCX) [file pone.0244436.s006.docx]

**S2 Table. Frequency of GATA3+ T cells.**

|  | **VC/S** | **TCS/S** | **VC/PR8** | **TCS/PR8** | **GATA3 FMO** |
| --- | --- | --- | --- | --- | --- |
| **BAL CD4+** | $ | $ | 0.096 ± 0.012 | 0.091 ± 0.022 | 0.081 |
| **BAL CD8+** | $ | $ | 0.005 ± 0.001 | 0.014 ± 0.004 | 0.017 |
| **Lung CD4+** | 0.99 ± 0.06 | 0.67 ± 0.08 | 0.24 ± 0.09 | 0.16 ± 0.05 | 0.018 |
| **Lung CD8+** | 0.42 ± 0.07 | 0.28 ± 0.021 | 0.08 ± 0.03 | 0.08 ± 0.01 | 0.26 |
| **Spleen CD4+** | 0.023 ± 0.003 | 0.022 ± 0.001 | 0.008 ± 0.002 | 0.007 ± 0.001 | 0.008 |
| **Spleen CD8+** | 0.017 ± 0.003 | 0.01 ± 0.003 | 0.013 ± 0.003 | 0.018 ± 0.003 | 0.014 |

Significance was assessed between all groups compared the VC/S control as determined by one-way ANOVA followed by a Dunnett’s post-test and by using an unpaired student’s t-test between the VC/PR8 and TCS/PR8 groups; no significant changes were found. $- not enough cells to accurately assess level of GATA3 expression.

$ not enough cells to accurately assess level of GATA3 expression.
