# Supplementary material for: Topical exposure to triclosan inhibits Th1 immune responses and reduces T cells responding to influenza infection in mice
Source: PLoS One. 2020 Dec 29;15(12):e0244436. doi: 10.1371/journal.pone.0244436 (PMC7771851; doi:10.1371/journal.pone.0244436)
Supplement: S3 Table — *s indicate significance against VC/PR8 control determined by an unpaired student’s t-test. * = P <0.05, ** = p < 0.01; n = 5 mice per group. (DOCX) [file pone.0244436.s007.docx]

**S3 Table. T-bet expression in activated and influenza responding T cells.**

|  | **BAL** | | **LLN** | | **Spleen** | |
| --- | --- | --- | --- | --- | --- | --- |
| **Tbet expression in:** | **VC/PR8** | **TCS/PR8** | **VC/PR8** | **TCS/PR8** | **VC/PR8** | **TCS/PR8** |
| **CD44^hi^ CD4+ (%)** | 15.66 ± 0.98 | 14.56 ± 2.04 | 2.70 ± 0.38 | 3.38 ± 0.74 | 1.982 ± 0.12 | 1.996 ± 0.21 |
| **CD44^hi^ CD4+ (#) x10^4^** | 2.49 ± 0.35 | 1.33 ± 0.28 * | 2.08 ± 0.35 | 2.58 ± 0.53” | 5.78 ± 0.53 | 5.23 ± 0.75 |
| **CD44^hi^ CD8+ (%)** | 23.04 ± 1.20 | 23.28 ± 2.85 | 5.60 ± 0.65 | 7.19 ± 1.47 | 4.192 ± 0.50 | 2.77 ± 0.15 * |
| **CD44^hi^ CD8+ (#) x10^4^** | 3.49 ± 0.36 | 2.67 ± 0.52 | 3.69 ± 0.64 | 5.22 ± 1.18 | 10.42 ± 0.81 | 7.43 ± 0.75 * |
| **Tet+ CD4+ (%)** | 15.14 ± 0.80 | 13.06 ± 1.95 | 6.65 ± 1.33 | 9.02 ± 2.56 | 1.794 ± 0.353 | 1.296 ± 0.287 |
| **Tet+ CD4+ (#)** | 1123 ± 458 | 319.5 ± 56.43 | 298.5 ± 77.85 | 392.4 ± 81.31 | 619.9 ± 151.7 | 232.8 ± 49.44 * |
| **Tet+ CD8+ (%)** | 20.42 ± 0.78 | 20.66 ± 2.69 | 18.58 ± 1.62 | 21.80 ± 6.02 | 13.28 ± 1.93 | 6.39 ± 0.97 * |
| **Tet+ CD8+ (#) x10^4^** | 0.63 ± 0.09 | 0.46 ± 0.12 | 0.66 ± 0.11 | 1.24 ± 0.61 | 1.869 ± 0.31 | 0.733 ± 0.089 ** |

*s indicate significance against VC/PR8 control determined by an unpaired student’s t-test. * = P <0.05, ** = p < 0.01; n= 5 mice per group.
